# PD-1 Affects the Immunosuppressive Function of Group 2 Innate Lymphoid Cells in Human Non-Small Cell Lung Cancer

**DOI:** 10.3389/fimmu.2021.680055

**Published:** 2021-06-14

**Authors:** Chunyi Shen, Chaojun Liu, Zhen Zhang, Yu Ping, Jingwen Shao, Yonggui Tian, Weina Yu, Guohui Qin, Shasha Liu, Liping Wang, Yi Zhang

**Affiliations:** ^1^ Biotherapy Center and Cancer Center, The First Affiliated Hospital of Zhengzhou University, Zhengzhou, China; ^2^ State Key Laboratory of Esophageal Cancer Prevention & Treatment, Zhengzhou University, Zhengzhou, China; ^3^ Department of Oncology, The First Affiliated Hospital of Zhengzhou University, Zhengzhou, China; ^4^ Henan Key Laboratory for Tumor Immunology and Biotherapy, Zhengzhou, China

**Keywords:** innate immune response, non-small cell lung cancer, programmed cell death protein 1, group 2 innate lymphoid cells, type 2 macrophage

## Abstract

**Background:**

There is increasing evidence that group 2 innate lymphoid cells (ILC2s) play an essential role in allergy and parasitic infection. However, the role of ILC2s in human lung cancer remains unclear.

**Methods:**

ILC2s from peripheral blood mononuclear cells (PBMCs) obtained from healthy donors (HDs) and non-small cell lung cancer (NSCLC) patients, and NSCLC tumor tissues were analyzed *via* multicolor flow cytometry. ILC2s or CD14^+^ cells were sorted by fluorescence-activated cell sorting. qPCR and flow cytometry were performed to assess the gene and protein expression of the indicated molecules. M1-like and M2-like macrophages were induced from CD14^+^ monocytes *in vitro*.

**Results:**

ILC2s were significantly more enriched in PBMCs and tumor tissues from NSCLC patients than in HDs. After screening for the main immune checkpoint molecules, we found that PD-1 was upregulated in ILC2s in NSCLC patients. Functionally, PD-1^high^ ILC2s from tumor tissues expressed higher levels of IL-4 and IL-13 regarding both mRNA and protein levels than PD-1^low^ ILC2s. Furthermore, PD-1^high^ ILC2s robustly boosted M2-like macrophage polarization *in vitro*, by secreting IL-4 and IL-13, while neutralization of IL-4 and IL-13 by antibodies abrogated M2-like macrophage polarization.

**Conclusion:**

ILC2s are enriched in NSCLC patients and upregulate PD-1 expression. Upregulation of PD-1 facilitates the immunosuppressive function of ILC2s. PD-1^high^ ILC2s enhance M2-like macrophage polarization by secreting IL-4 and IL-13. PD-1 acts as a positive regulator of the immunosuppressive function of ILC2s in human NSCLC.

## Introduction

Generally, the family of innate lymphoid cells (ILCs) comprises two cardinal lineages: cytotoxic or killer ILCs (cNK cells) and helper-like ILCs (ILC1s, ILC2s, and ILC3s) (1–3). The transcriptional programs and effector functions of various ILC populations resemble those of CD4^+^ helper T cell subsets (4). For example, specific transcriptional factor GATA3 controls the fate and maintenance of Th2 cells and ILC2s, both of which produce mainly type 2 cytokines, such as interleukin (IL)-4 and IL-13 (5–7).

Inflammatory processes play major roles in diverse cancers, including lung cancer, and facilitate tumor initiation, progression, and metastasis (8, 9). An increasing number of studies have demonstrated that ILC2s played an essential role in the inflammatory processes that underlie allergy and parasitic infection (10–12). Some studies have investigated the role of ILC2s in tumor settings. In a mouse model of melanoma, ILC2s were found to inhibit NK cell activation and cytotoxicity, thereby further aggravating tumor progression (13). In addition, ILC2s enhanced the accumulation of myeloid-derived suppressor cells (MDSCs) by upregulating IL-13, which was involved in the recurrence of bladder cancer (14). In a 4T1 breast cancer mouse model, Ivan et al. reported that IL-5 and IL-13-expressing ILCs hastened tumor progression and metastasis; unfortunately, the authors did not confirm whether the ILCs expressing IL-5 and IL-13 were ILC2s (15). Most recently, Trabanelli et al. summarized the evidence from research about ILC2s in tumors and found that the majority of studies declared that ILC2s functioned as a pro-tumor factor by activating and/or recruiting other stromal cells (16). Lung cancer, the commonest of which is non-small cell lung cancer (NSCLC), has the highest incidence and mortality rate among various tumors worldwide (17). It has been demonstrated that the frequency of ILC2s increases in patients with lung cancer, which promotes lung metastases and mortality by inhibiting NK cell cytotoxic function and enhancing regulatory T cell (Treg) immunosuppressive manner (18–20). However, little is known about the PD-1 expression of ILC and the role of PD-1 in ILC2s in human lung cancer.

In the present study, we found that ILC2s were enriched in peripheral blood and tumor tissues of patients with NSCLC, and showed a more powerful immunosuppressive function than ILC2s obtained from healthy donors (HDs). PD-1 was upregulated in ILC2s obtained from NSCLC patients and corelated with high expression of IL-4 and IL-13. In tumor microenvironment, tumor-associated macrophages (TAMs) have two states of polarization (M1 and M2) according to different function and response to stimuli (21). Furthermore, the PD-1^high^ ILC2s could increase M2-like macrophage polarization *via* IL-4 and IL-13. Our findings suggest that PD-1 plays an important role in the immunosuppressive function of ILC2s in human NSCLC.

## Materials and Methods

### Patients and Healthy Donors

Peripheral blood samples and fresh tumor tissues were obtained from 70 patients with NSCLC who underwent surgical resection at the First Affiliated Hospital of Zhengzhou University. None of the patients had received chemotherapy or radiotherapy before sampling. Written informed consent was obtained from each subject enrolled in this study. The study protocol was approved by the Ethics Committee of the First Affiliated Hospital of Zhengzhou University. Details of the clinicopathologic features of these patients are summarized in **Supplementary Table S1**. The peripheral blood samples from 20 HDs were used as control.

### Acquisition of Peripheral Blood Mononuclear Cells and Tumor Tissue Single Cell Suspension

Peripheral blood samples were subjected to Ficoll–Paque density gradient centrifugation at 2,500 rpm for 25 mins. Peripheral blood mononuclear cells (PBMCs) were extracted and washed twice with sterile phosphate buffered saline (PBS) (cat# P1020; Solarbio, China). Fresh tumor tissues were dissociated into single cell suspensions utilizing the human tumor dissociation kit (cat# 130-095-929; Miltenyi, Germany) following the manufacturer’s instructions. Tumor tissue single cell suspensions were filtered using a 70 μm cell strainer and then washed twice with PBS for subsequent flow cytometry testing or cell sorting.

### Flow Cytometry Analysis

Human ILC2s were identified utilizing the CD45^+^Lin^−^CD127^+^CRTH2^+^ gate strategy described in previous studies (22–24). Cell surface molecules were stained by incubating PBMCs and the tumor tissue single cell suspension with fluorochrome-conjugated primary antibodies away from light for 20 mins at 4°C. Intracellular staining of IL-4, IL-13, p-AKT, p-S6, and p-STAT5 was executed as described in a previous study (25). In brief, after cell surface staining, cells were fixed with 4% paraformaldehyde, permeabilized by perm wash buffer, and stained with anti-IL-4 and anti-IL-13 for 20 min at 4°C in the dark. For detection of p-AKT, p-S6, and p-STAT5, the cells were first stained with the primary antibodies following surface staining, fixation and permeabilization, and then stained with the secondary antibodies. The cells were tested and analyzed utilizing the flow cytometry system (Canto II; BD Biosciences, USA).

The following anti-human monoclonal antibodies were used for flow cytometry [all purchased from BioLegend (San Diego, CA, USA) except when indicated]: FITC anti-human CD45 (cat# 368508; clone: 2D1), PerCP-Cy5.5 anti-human CD45 (cat# 368506; clone: 2D1), PerCP-Cy5.5 anti-human CD127 (IL7R) (cat# 351322; clone: A019D5), PE anti-human CD127 (cat# 351316; clone: A019D5), PE anti-human CD279 (PD-1) (cat# 329906; clone: EH12.2H7), PE-Cy7 anti-human CD279 (cat# 329918; clone: EH12.2H7), APC-Cy7 anti-human CD294 (CRTH2) (cat# 350113; BM16), PE anti-human CD117(c-kit) (cat# 313204; clone: 104D2), APC anti-human lineage cocktail [cat# 348803; clone: UCHT1 (CD3), HCD14 (CD14), 3G8 (CD16), HIB19 (CD19), 2H7 (CD20), and HCD56 (CD56)], PE anti-human IL-4 (cat# 500810; clone: MP4-25D2), PE anti-human IL-13 (cat# 501903; clone: JES10-5A2). The primary antibodies against phosphorylated AKT (p-AKT, Ser473, clone: D9E, cat # 4060T, dilution 1:100), phosphorylated S6 (p-S6, Ser235/236 clone: D57.2.2E, cat # 4858T, dilution 1:25), and phosphorylated STAT5 (p-STAT5, Tyr694, clone D47E7, cat # 4322T, dilution 1:200) were purchased from Cell Signaling Technology (Boston, USA). The secondary antibodies were FITC Donkey anti-rabbit IgG (cat# 406403, clone: Poly4064; BioLegend) and PE Donkey anti-rabbit IgG (cat# 406421, clone: Poly4064; BioLegend).

### Cell Isolation, RNA Extraction, and Reverse Transcription

ILC2s (CD45^+^Lin^−^CD127^+^CRTH2^+^), PD-1^high^ ILC2s, and PD-1^low^ ILC2s were sorted from PBMCs or tumor tissue single cell suspensions by fluorescence-activated cell sorting (FACS) (Moflo XDP; Beckman, USA). Total RNA was extracted from 100–1000 sorted ILC2s cells, PD-1^high^ ILC2s, PD-1^low^ ILC2s, or CD14^+^ cells, and cDNA was simultaneously generated using the single cell sequence specific amplification kit (cat# P621-01; Vazyme Biotech Co. Ltd., China) following the manufacturer’s instructions.

### Quantitative Real-Time Polymerase Chain Reaction

Quantitative real-time polymerase chain reaction (qRT-PCR) was performed in with the CFX96 Touch Real-Time PCR Detection System (Bio-Rad, USA) using the ChamQ SYBR Color qPCR Master Mix (cat# Q411-02; Vazyme, China) following the manufacturer’s instructions. Target genes were amplified under the following conditions: 95°C for 5 min and 40 cycles of 95°C for 30 sec, 60°C for 30 sec. *GAPDH* was used as the internal control and relative gene expression levels were ascertained by the 2^-ΔΔCt^ method followed by logarithmic transformation to the base 2. The primers of the target genes are listed in **Supplementary Table S2**.

### Macrophage Polarization *In Vitro*

CD14^+^ monocytes were sorted from PBMCs of HDs *via* magnetic-activated cell sorting (cat# 130-118-906; Miltenyi Biotec, Germany) according to the manufacturer’s instructions. Sorted PD-1^high^ ILC2s and PD-1^low^ ILC2s were cultured in RPMI 1640 complete medium containing 10% fetal bovine serum (cat# S711-001; LONSERA, Australia) and 1% penicillin-streptomycin solution (cat# P1400; Solarbio, China) with 10 IU/ml of recombinant human IL-2 (cat# 200-02; PeproTech, USA) and 20 ng/ml of IL-7 (cat# 200-07; PeproTech, USA) for 48 h, after which the supernatant was harvested. Sorted CD14^+^ monocyte cells obtained from HD PBMCs were cultured to induce macrophages in complete RPMI 1640 culture medium and 100 ng/ml recombinant human M-CSF (rhM-CSF; cat# 300-25; PeproTech, USA) in a 24-well plate with 1 × 10^6^ cells per well for 7 days (26). Then, the cells were treated with 100 ng/ml of LPS and 20 ng/ml recombinant human IFN-γ (rhIFN-γ; cat# 300-02; PeproTech, USA) (for M1-like macrophages) or 20 ng/ml recombinant human IL-4 (rhIL-4; cat# 200-04; PeproTech, USA) (for M2-like macrophages) or PD-1^low^ ILC2s culture supernatant or PD-1^high^ ILC2s culture supernatant for 18 h. With respect to the IL-4 and IL-13 blockade assay, CD14^+^ monocyte cells sorted from HD PBMCs were cultured in RPMI 1640 complete medium and 100 ng/ml of rhM-CSF (PeproTech) in a 24-well plate of 1×10^6^ cells per well for 7 days. Then, they were divided into four groups and the following were added: the solute, anti-IL-4 antibody (cat# 500838, clone: MP4-25D2; BioLegend), anti-IL-13 antibody (cat# 501910, clone: JES10-5A2; BioLegend), and anti-IL-4 antibody combined with anti-IL-13 antibody. Two hours later, 100 ng/ml of LPS and PD-1^high^ ILC2s culture supernatant were added and cultured for an additional 18 hours. The cells were harvested for flow cytometry testing, RNA extraction, and qPCR. The final concentrations of IL-4 and IL-13 antibodies in the medium were 1 μg/ml and 500 ng/ml, respectively.

### Statistical Analysis

The results of flow cytometry were analyzed using FlowJo software version X (BD, USA). Other analyses were performed using Prism software version 7.0 (GraphPad Software Inc, USA). The results are presented as means with their standard errors. Two-group comparison was performed using the two-tailed student’s *t*-test. Comparisons among more than two groups were performed *via* one-way analysis of variance with Tukey’s *post hoc* tests. Analysis items with *P* value less than 0.05 were considered statistically significant.

## Results

### Identification and Distribution of ILC2s Among HDs and NSCLC Patients

**Figure 1:**
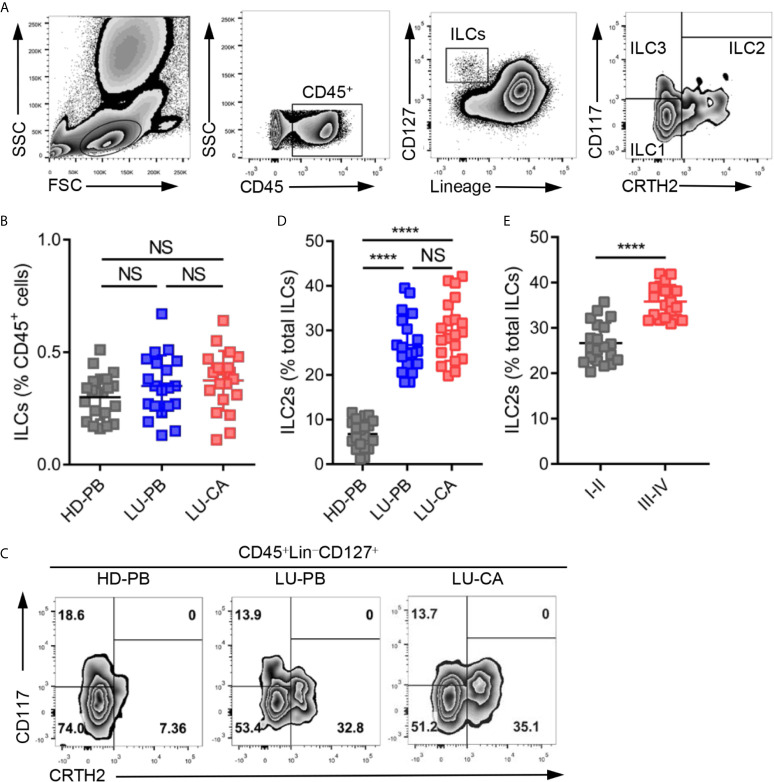
Identification and distribution of group 2 innate lymphoid cells in healthy donors and NSCLC patients. **(A)** The representative gating strategy of human ILCs (CD45^+^Lin (CD3, CD14, CD16, CD19, CD20, CD56)^-^CD127^+^). ILC1s: CD45^+^Lin^-^CD127^+^CRTH2^-^CD117^-^; ILC2s: CD45^+^Lin^-^CD127^+^CRTH2^+^; ILC3s: CD45^+^Lin^-^CD127^+^CRTH2^-^CD117^+^. **(B)** The proportions of total ILCs among CD45^+^ cells from HD-PB (PBMCs from healthy donors, n=20), LU-PB (PBMCs from NSCLC patients, n=20), and LU-CA (tumor single cell suspension from NSCLC patients, n=20). **(C)** The representative results of ILC subset distribution in HD-PB, LU-PB, and LU-CA samples assessed *via* flow cytometry. **(D)** The proportions of ILC2s among total ILCs in HD-PB, LU-PB, and LU-CA (for each group, n=20). **(E)** The proportions of ILC2s among total ILCs of LU-CA at different stages (for each group, n=20). NS, not significant; ****P<0.0001. In **(B, D)**, *P* values were calculated by one-way ANOVA and *post hoc* comparisons were performed *via* Tukey’s test. In **(E)**, *P* values were obtained in a non-paired two-tailed student’s *t*-test.

Total ILCs and ILC2s were identified as CD45^+^Lin^−^CD127^+^ cells and CD45^+^Lin^−^CD127^+^CRTH2^+^ cells, respectively, in line with previous studies (22–24). The representative gating strategies are shown in **Figure 1A**. The proportions of total ILCs among CD45^+^ cells were measured in HDs and NSCLC patients. There was no significant difference in the proportions of ILCs among CD45^+^ cells obtained from HDs and NSCLC patients (**Figure 1B**, **Supplementary Figure 1A**). Next, the proportions of three ILC subsets were assessed, the representative results of which are shown in **Figure 1C**. ILC2s constituted a much higher proportion of total ILCs in PBMCs (*P*<0.0001) and tumor tissues (*P*<0.0001) obtained from NSCLC patients than in PBMCs obtained from HDs (**Figure 1D**, **Supplementary Figure 1B**). However there was no significant difference between tumor tissues and adjacent tissues (**Supplementary Figure 1C**). NSCLC patients also showed remarkably decreased and increased the levels of ILC1s and ILC3s, respectively (**Supplementary Figures 1D, E**). ILC2s were selectively enriched in NSCLC patients, which drove us to explore the correlation between ILC2 levels and tumor stages among NSCLC patients. The proportions of ILC2s among total ILCs in patients with stage III–IV disease were significantly higher than those in patients with stage I–II disease (**Figure 1E**, **Supplementary Figure 1F**). In addition, ILC1s were decreased in patients with stage III–IV disease, and there was no difference in the level of ILC3s between the two groups (**Supplementary Figures 1G, H**). Together, these findings indicated that although the proportion of total ILCs among CD45^+^ cells showed no difference, ILC2s were enriched in both PBMCs and tumor tissues obtained from NSCLC patients and ILC1s decreased in NSCLC patients.

### Genetic Expression of Key Phenotypic and Functional Molecules in ILC2s

**Figure 2:**
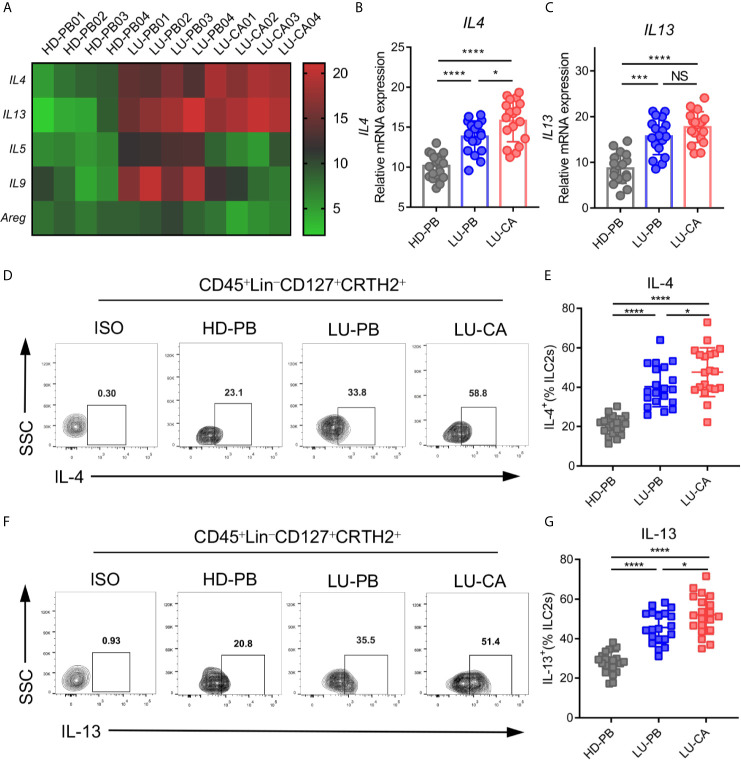
mRNA and protein expression of key functional molecules of ILC2s. **(A–C)** ILC2s were marked as CD45^+^Lin^-^CD127^+^CRTH2^+^ and sorted *via* FACS. **(A)** A heatmap showing representative results of mRNA expression of the key functional molecules of ILC2s obtained from HD-PB, LU-PB, and LU-CA samples. **(B, C)** The relative mRNA expressions of *IL4* **(B)** and *IL13* **(C)** in ILC2s from HD-PB, LU-PB, and LU-CA samples (for each group, n=18). **(D–G)** Protein expression of IL-4 **(D, E)** and IL-13 **(F, G)** in ILC2s from HD-PB, LU-PB, and LU-CA samples assessed *via* flow cytometry (for each group, n=20). HD-PB: PBMCs from healthy donors, LU-PB: PBMCs from NSCLC patients, and LU-CA: tumor single cell suspension from NSCLC patients. NS, not significant; *P<0.05, ***P<0.001, ****P<0.0001. P values were calculated by one-way ANOVA and *post hoc* comparisons were performed *via* Tukey’s test.

Since ILC2s increased in NSCLC patients, we investigated gene expression in ILC2s. ILC2s were sorted from HD PBMCs, and PBMCs and tumor tissues of NSCLC patients *via* FACS. Total RNA extraction and cDNA generation were performed. Gene expression profiles of key phenotypic and functional molecules of ILC2s were tested by qPCR. There were no difference between NSCLC patients and HDs regarding phenotypic gene expressions of *IL7R*, *CRTH2*, and *ST2*. PBMCs obtained from NSCLC patients, but not tumor tissues, showed increased expression of *KIT*(*CD117*) compared with those from HDs (**Supplementary Figure 2A**). Next, the genetic expression of major functional molecules of ILC2s (*IL4*, *IL5*, *IL9*, *IL13*, and *AREG*) (23) were tested (**Figure 2A**). *IL4* and *IL13* were highly expressed in ILC2s obtained from tumor tissues (*IL4*, *P*<0.0001; *IL13*, *P*<0.0001) and PBMCs (*IL4*, *P*<0.0001; *IL13*, *P*<0.0001) obtained from NSCLC patients than those obtained from HDs (**Figures 2B, C**). In addition, *IL4* expression in ILC2s obtained from tumor tissues was higher than NSCLC PBMCs (**Figure 2B**), while there was no distinction in *IL13* expression between these two groups (**Figure 2C**). Intriguingly, the expression of *IL5* and *IL9* was increased in ILC2s from PBMCs from NSCLC patients, but not in ILC2s from tumor tissues, compared with HD-derived PBMCs (**Supplementary Figure 2B**). This result indicated that the expression of *IL5* and *IL9* in ILC2s might not be affected by tumor site. In addition, there was no difference in *AREG* expression between NSCLC patients and HDs (**Supplementary Figure 2B**). These results showed that *IL4* and *IL13* were upregulated in ILC2s obtained from both PBMCs and tumor tissues of NSCLC patients. Furthermore, the protein levels of IL-4 and IL-13 were confirmed by multi-color flow cytometry. As expected, IL-4 and IL-13 levels were highly increased in ILC2s from PBMCs (IL-4, *P*<0.0001; IL-13, *P*<0.0001) (**Figures 2D, E**) and tumor tissues (IL-4, *P*<0.0001; IL-13, *P*<0.0001) (**Figures 2F, G**) of NSCLC patients than in ILC2s from HD PBMCs. We also found that the protein levels of IL-4 and IL-13 of ILC2s from NSCLC tumor tissues were higher than those from NSCLC PBMCs. This implied that certain factors in the tumor microenvironment might further boost the upregulation of IL-4 and IL-13 in tumor tissues. Taken together, these results demonstrated that both IL-4 and IL-13 were upregulated in ILC2s of NSCLC patients, particularly in ILC2s derived from tumor tissues.

### Increased Expression of PD-1 on ILC2s in NSCLC Patients

**Figure 3:**
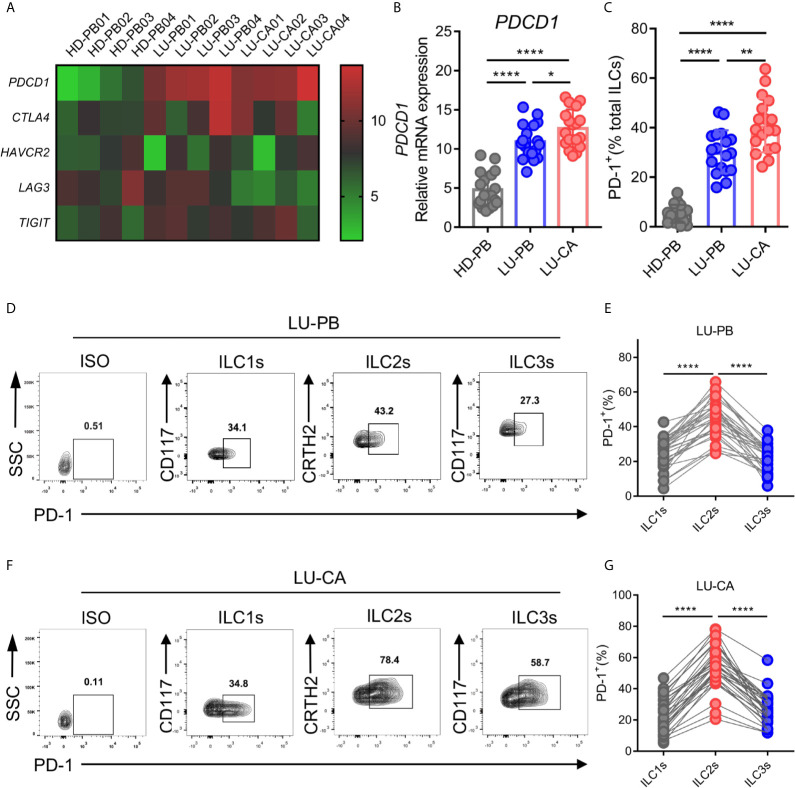
mRNA and protein expression of immune checkpoint molecules of ILC2s. **(A–C)** The samples of ILC2s used were the same as in **Figure 2**. **(A)** The heatmap showing relative mRNA expression of *PDCD1*, *CTLA4*, *HAVCR2* (encoding TIM3), *LAG3*, and *TIGIT* on ILC2s in HD-PB, LU-PB, and LU-CA samples. **(B)** The relative mRNA expression of *PDCD1* on ILC2s from HD-PB, LU-PB, and LU-CA samples (for each group, n=18). **(C)** The protein expression of PD-1 on total ILCs obtained from HD-PB, LU-PB, and LU-CA samples (for each group, n=18). **(D–G)** The representative results and statistical results of PD-1 protein expression on ILCs subsets in LU-PB **(D, E)** and LU-CA **(F, G)** assessed by flow cytometry (for each group, n=30). *P<0.05, **P<0.01, ****P<0.0001. P values were calculated by one-way ANOVA and *post hoc* comparisons were performed *via* Tukey’s test.

A previous study demonstrated that PD-1 expression plays an important role in ILC2 development and function (27). Thus, we explored the gene expression profile of *PDCD1* (encoding PD-1) as well as other cardinal immune checkpoint molecules in ILC2s from NSCLC patients. ILC2s were sorted from HD-derived PBMCs and NSCLC patient-derived PBMCs and tumor tissues *via* FACS, and then subjected to RNA extraction and qPCR. The representative data in a heatmap are shown in **Figure 3A**. *PDCD1* was obviously upregulated in ILC2s obtained from PBMCs (*P*<0.0001) and tumor tissues (*P*<0.0001) of NSCLC patients compared with ILC2s from HDs (**Figure 3B**). Moreover, *PDCD1* expression was increased in ILC2s obtained from tumor tissues compared with ILC2s obtained from NSCLC PBMCs (**Figure 3B**). There were no differences in the expressions of other immune checkpoint genes (*CTLA4*, *TIM3*, and *TIGIT*), except for *LAG3* whose expression in ILC2s from tumor tissues was lower than that in PBMCs from HDs or NSCLC patients (**Supplementary Figure 3A**). PD-1 on total ILCs obtained from PBMCs (*P*<0.0001) and tumor tissues (*P*<0.0001) of NSCLC patients were significantly upregulated compared with those on ILCs obtained from HD PBMCs (**Figure 3C**). In addition, PD-1 expression on ILCs from tumor tissues was higher than that on ILCs from PBMCs of NSCLC patients (**Figure 3C**). Next, we investigated the levels of PD-1 on three subsets of ILCs from PBMCs and tumor tissues of NSCLC patients. The proportions of PD-1 expression in ILC2s were much higher than those in ILC1s or ILC3s in both PBMCs (**Figures 3D, E**) and tumor tissues (**Figures 3F, G**) from NSCLC patients. In addition, we also observed that the proportions of PD-1 expression in ILC2s were increased in tumor tissues than in adjacent tissues though it did not show significant difference in frequency of ILC2s (**Supplementary Figures 1C, 3B**). Thus, PD-1 was highly expressed on ILC2s obtained from NSCLC patients both in terms of mRNA level and protein level, indicating that PD-1 may function as a regulatory factor, to some degree, in ILC2s from NSCLC patients.

### Higher Expression of IL-4 and IL-13 in PD1^high^ ILC2s From NSCLC Patients

**Figure 4:**
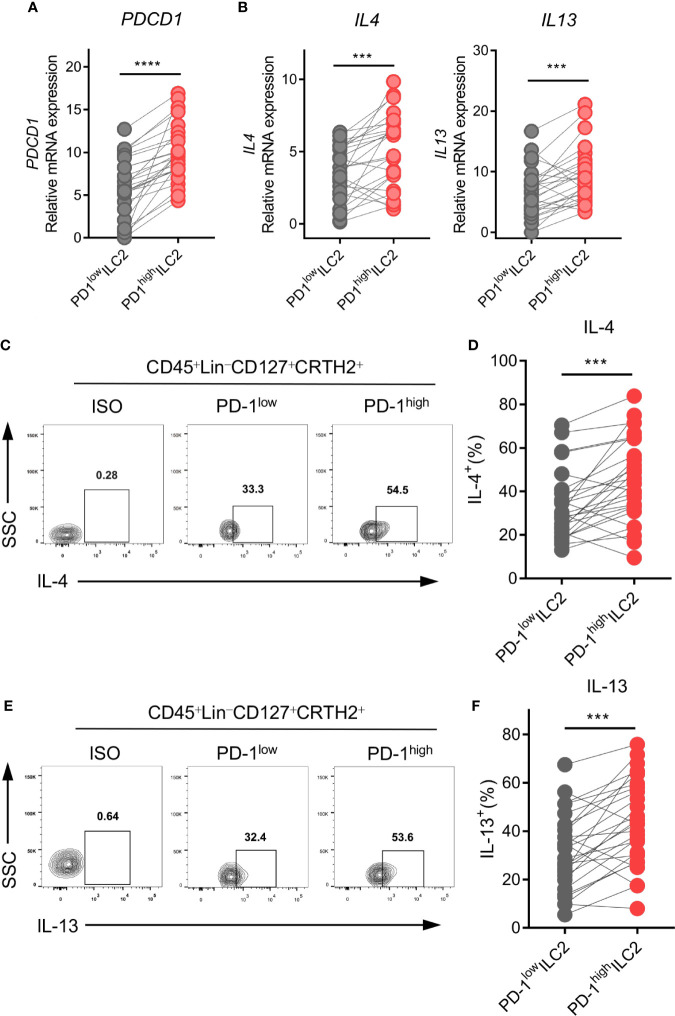
PD-1^high^ ILC2s expressed higher levels of IL-4 and IL-13. PD-1^high^ ILC2s and PD-1^low^ ILC2s were marked as CD45^+^Lin^-^CD127^+^CRTH2^+^PD-1^high^ and CD45^+^Lin^-^CD127^+^CRTH2^+^ PD-1^low^, respectively, and sorted by FACS. **(A, B)** The gene expressions of *PDCD1* (encoding PD-1) **(A)**, *IL4*, and *IL13* **(B)** were upregulated in the PD-1^high^ group compared with the matched PD-1^low^ group (n=26). **(C–F)** The representative results and statistical results of IL-4 **(C, D)** and IL-13 **(E, F)** protein expression according to the different PD-1 levels assessed by flow cytometry (for each group, n=26). Each dot represents one sample. ***P<0.001, ****P<0.0001. P values were calculated using a paired two-tailed student’s *t*-test.

Based on the finding that PD-1 was upregulated on ILC2s in NSCLC patients, we hypothesized that PD-1 might play a role in the regulation of ILC2 function. PD-1^high^ and PD-1^low^ ILC2s from human NSCLC tumor tissues were sorted *via* FACS. We then assessed the genetic expression of ILC2 phenotype, function, and checkpoint molecules *via* qPCR. As expected, PD-1^high^ ILC2s showed obviously higher *PDCD1* expression than PD-1^low^ ILC2s (**Figure 4A**). The expression of *CRTH2* and *ST2* was upregulated in PD-1^high^ ILC2s compared with PD-1^low^ ILC2s, while no difference was found in the expression of *IL7R* and *KIT*(*CD117*) (**Supplementary Figure 4A**). Furthermore, the immune checkpoint molecules *CTLA4*, *HAVCR2* (*TIM3*), and *LAG3* showed comparable levels of gene expression according to the different PD-1 levels, except for *TIGIT* which was highly expressed in PD-1^high^ ILC2s (**Supplementary Figure 4B**).

Regarding the functional molecules of ILC2s, PD-1^high^ ILC2s in tumor tissues expressed much higher levels of *IL4* and *IL13* than did PD-1^low^ ILC2s in terms of mRNA level (**Figure 4B**). However, there were no differences in the expression of other functional molecules (*IL5*, *IL9*, and *AREG*) (**Supplementary Figure 4C**). Next, we performed flow cytometry to confirm the protein levels of IL-4 and IL-13 in ILC2s. As expected, levels of IL-4 (**Figures 4C, D**) and IL-13 (**Figures 4E, F**) were higher in PD-1^high^ ILC2s than in PD-1^low^ ILC2s. Taken together, the upregulation of PD-1 in ILC2s indicated more powerful function, and particularly induced the upregulation of type 2 cytokines (IL-4 and IL-13).

### Upregulation of PD-1 Correlated With the AKT-S6 Signaling Pathway Activation in ILC2s Obtained From NSCLC Patients

**Figure 5:**
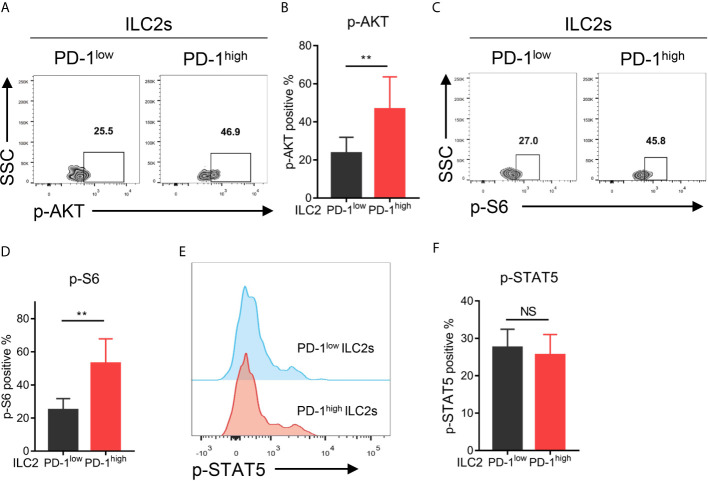
Upregulation of the PD-1 activated AKT-S6 signaling pathway in ILC2s obtained from NSCLC tumor tissues. **(A, B)** Phosphorylated AKT level was increased in PD-1^high^ ILC2s compared with matched PD-1^low^ ILC2s (n=6, *P*=0.0029). **(C, D)** Phosphorylated S6 level was increased in PD-1^high^ ILC2s compared with matched PD-1^low^ ILC2s (n=6, *P*=0.0055). **(E, F)** There was no difference in the levels of phosphorylated STAT5 between PD-1^high^ ILC2s and PD-1^low^ ILC2s (n=6). NS, not significant; **P<0.01. P values were calculated using a paired two-tailed student’s *t*-test.

To investigate the downstream signaling pathway of PD-1 in ILC2s, we assessed the phosphorylation levels of the key molecules within the PD-1 signaling pathway (such as AKT and S6) as previously reported (28, 29) *via* flow cytometry. As a result, PD-1^high^ ILC2s upregulated phosphorylated AKT (p-AKT) compared with PD-1^low^ ILC2s (**Figures 5A, B**). Similarly, the proportion of phosphorylated S6 (p-S6) in PD-1^high^ ILC2s was significantly increased compared with that in PD-1^low^ ILC2s (**Figures 5C, D**). These findings suggested that PD-1 upregulation activated its downstream signaling molecules in ILC2s of NSCLC patients. A previous study showed that phosphorylated STAT5 (p-STAT5) was a key downstream molecule of the PD-1 signaling pathway in ILC2s and inhibited IL-5 and IL-13 expression in the context of infection with *Nippostrongylus brasiliensis* (30). This drove us to explore whether STAT5 plays a role in the regulation of cytokine secretion in ILC2s of NSCLC patients. While, we did not find a significant difference in p-STAT5 levels between PD-1^high^ ILC2s and PD-1^low^ ILC2s (**Figures 5E, F**), suggesting that p-STAT5 played a marginal role in the functional regulation of PD-1 on ILC2s in human NSCLC tumors. Thus, PD-1 upregulation on ILC2s scarcely effects p-STAT5 signaling and may activate the AKT-S6 signaling pathway in NSCLC patients.

### PD1^high^ ILC2s Enhance the Polarization of M2-Like Macrophages From Monocytes

**Figure 6:**
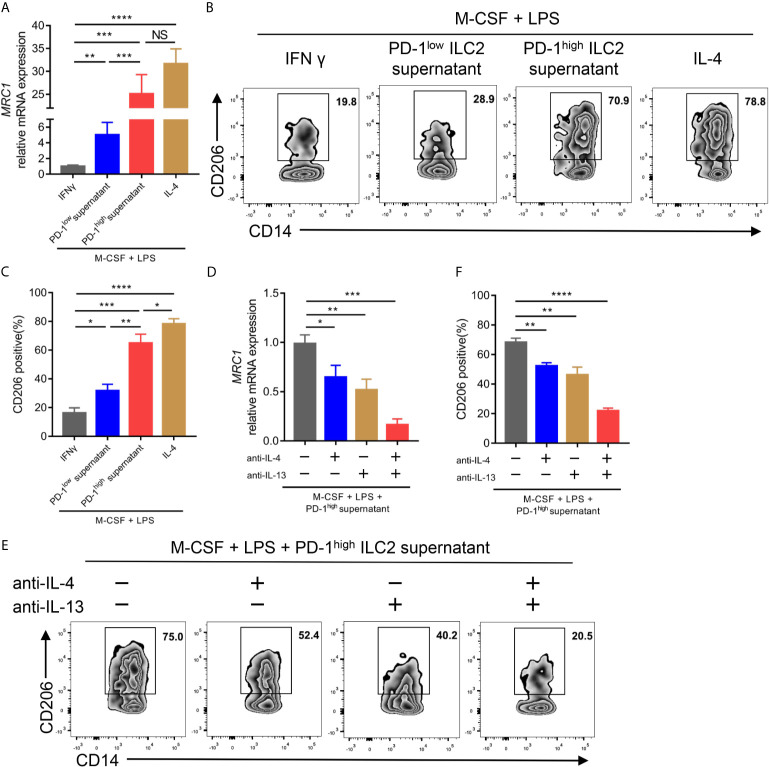
PD-1^high^ ILC2s enhanced the polarization of M2-like macrophages by upregulating IL-4 and IL-13 *in vitro*. **(A–C)** Sorted CD14^+^ monocytes from HD PBMCs were treated with recombinant human M-CSF for seven days, and then mixed with LPS and IFNγ (for M1-like macrophages) or IL-4 (for M2-like macrophages) or PD-1^high^ ILC2s culture supernatant or PD-1^low^ ILC2s culture supernatant for an additional 18 hours. The CD14^+^ cells were harvested for qPCR and flow cytometry. **(A)** PD-1^high^ ILC2 culture supernatant enhanced *MRC1* (encoding CD206, a well-known M2 macrophage marker) mRNA expression compared with PD-1^low^ ILC2 culture supernatant treated with CD14^+^ cells. **(B, C)** The CD206 protein expression was assessed *via* flow cytometry, the representative results **(B)** and statistical results **(C)** (n=6) of which are shown. **(D–F)** CD14^+^ monocytes sorted from HD PBMCs were treated with recombinant human M-CSF for seven days and divided into four groups to which the following were added: the solute, anti-IL-4 antibody, anti-IL-13 antibody, and a combination of anti-IL-4 and anti-IL-13 antibodies, respectively. Two hours later, LPS and PD-1^high^ ILC2 supernatant were added to all groups and cultured for another 18 hours. **(D)** Anti-IL-4 and/or anti-IL-13 antibodies decreased the mRNA expression of *MRC1*. **(E, F)** The levels of CD206 were diminished after IL-4 and/or IL-13 blockade testing by flow cytometry, the representative results **(E)** and statistical results **(F)** (n=5) of which are shown. NS, not significant; *P<0.05, **P<0.01, ***P<0.001, ****P<0.0001. P values were calculated by one-way ANOVA and *post hoc* comparisons were performed *via* Tukey’s test.

Previous studies have demonstrated that IL-4 and IL-13, two canonical type 2 cytokines, could mediate M2 macrophage polarization (31, 32). Considering the upregulation of IL-4 and IL-13 in PD-1^high^ ILC2s, we assessed the effect of ILC2 culture supernatant on the process of CD14^+^ monocyte polarization to M2-like macrophages *in vitro*. We found that the culture supernatant of PD-1^high^ ILC2s enhanced the gene expression of mannose receptor C-type 1 (*MRC1*; encoding CD206, a well-known marker for M2 macrophage) in terms of both mRNA level (**Figure 6A**) and protein level (**Figures 6B, C**) compared with matched PD-1^low^ culture supernatant. The expression of M1 macrophage related genes (*TNF*, *IL6*, and *CCL5*) and M2 macrophage related genes (*TGFB1*, *CCL18*, and *ARG1*) (26) was also assessed. As expected, the expression of *TNF*, *IL6*, and *CCL5* in CD14^+^ cells treated with PD-1^high^ ILC2s supernatant was obviously decreased compared with matched PD-1^low^ ILC2s supernatant treated CD14^+^ monocytes or M1-like macrophages (**Supplementary Figure 5A**). On the contrary, the expressions of M2 macrophage related genes (*TGFB1*, *CCL18*, and *ARG1*) in CD14^+^ monocytes treated with PD-1^high^ ILC2s supernatant were evidently increased compared with matched PD-1^low^ ILC2s supernatant treated CD14^+^ monocytes or M1-like macrophages (**Supplementary Figure 5B**). These results suggested that some soluble molecules in the PD-1^high^ ILC2s culture supernatant boosted the polarization of M2-like macrophages from CD14^+^ monocytes. To assess whether elevated levels of IL-4 and IL-13 contributed to this phenomenon, we applied IL-4 and/or IL-13 neutralizing antibodies prior to treatment with PD-1^high^ ILC2s supernatant. The gene expression of *MRC1* in PD-1^high^ ILC2s supernatant was partially inhibited by anti-IL-4 antibody or anti-IL-13 antibody alone, and was almost abolished by a combination of anti-IL-4 and anti-IL-13 antibodies (**Figure 6D**). Consistent results of corresponding protein levels were confirmed *via* flow cytometry (**Figures 6E, F**). Furthermore, the expression of M2 macrophage related genes (*TGFB1*, *CCL18*, and *ARG1*) in CD14^+^ cells treated with PD-1^high^ ILC2s supernatant was partially downregulated by anti-IL-4 antibody or anti-IL-13 antibody alone, and further inhibited by a combination of anti-IL-4 and anti-IL-13 antibodies (**Supplementary Figure 5C**). These results indicated that IL-4 and IL-13 played a role in the process by which PD-1^high^ ILC2s induce the polarization of M2-like macrophages from CD14^+^ monocytes. Thus, we demonstrated that PD-1^high^ ILC2s enhanced the polarization of M2-like macrophages from CD14^+^ monocytes through the upregulation of IL-4 and IL-13, *in vitro*.

## Discussion

ILCs, except for canonical NK cells, were new groups of lymphoid cells that were identified two decades ago (23). Since then, many studies on ILCs have been conducted in the fields of infectious and allergic diseases and, on a smaller scale, in tumors. Despite constituting a considerable proportion of immune cells in infection and allergy, the proportions of ILCs in tumors are relatively low, making it more difficult to investigate their roles in tumors. To our best knowledge, no published study has focused on ILC2s in human NSCLC to date. Thus, the present study assessed the distribution and functional features of ILC2s, as well as PD-1 expression and function on ILC2s in NSCLC patients.

We found no difference in the proportion of total ILCs in CD45^+^ cells between HDs and NSCLC patients; however, the proportions of ILC2s in bulk ILCs obtained from PBMCs and tumor tissues of NSCLC patients were significantly higher than those obtained from HD PBMCs. Similarly, Salimi et al. reported that ILC2s were enriched in human breast cancer tissues compared with tumor-adjacent tissues (33).

Moreover, we found that ILC2s in NSCLC patients expressed high levels of IL-4 and IL-13, which are classical type 2 cytokines, and might execute immunosuppressive function in human NSCLC. Similarly, a previous study reported that ILC2s contributed to melanoma progression by inhibiting NK cell activation and cytotoxicity in a mouse model, thereby functioning as an immunosuppressive factor (13). Another study showed that ILC2s facilitated tumor recurrence *via* the accumulation of MDSC by upregulating IL-13 among patients with bladder cancer (14).

In a murine model of metastatic lung tumor, a deficiency of ILC2s significantly boosted tumor growth and metastasis, indicating that ILC2s might function as an anti-tumor factor in murine lung cancer (34). However, the approach to deleting ILC2s using RORα^-/-^ mice in this study could unavoidably impair the development and function of some other cells, which probably biased the research outcome. Together, our findings indicated that ILC2s were enriched in human NSCLC and might function as immunosuppressive cells *via* the upregulation of the type 2 cytokines IL-4 and IL-13.

In the area of adaptive immunity, PD-1 predominantly causes T cell function inhibition and exhaustion (35, 36). The downregulation of PD-1 may restore the anti-tumor function of CD8+ T cells (37). Targeting the glycosylation of PD-1 enhances the cytotoxicity of chimeric antigen receptor T cells in tumor milieu (38). Regarding PD-1 on ILC2s, Yong et al. reported that PD-1 was upregulated on ILC2 progenitor cells and mature ILC2s in lungs after influenza infection and that blocking PD-1 on PD-1^high^ ILC2s using anti-PD-1 antibody dramatically reduced cytokine (such as IL-13) production in the context of infection (27), indicating that PD-1 plays an indispensable role in ILC2 development and function. In addition, a previous study reported that PD-1 was exclusively expressed by ILC2s compared with ILC1s or ILC3s in mice (30). With respect to tumor settings, Moral et al. reported that PD-1 was highly expressed on tumor-infiltrating ILC2s in human and mouse pancreatic ductal adenocarcinoma (PDAC) (39). Similarly, we found that PD-1 was highly expressed in ILC2s compared with matched ILC1s or ILC3s in NSCLC patients. To explore the role of PD-1 upregulation in ILC2s, we sorted PD-1^high^ ILC2s and PD-1^low^ ILC2s from NSCLC tumor tissues by FACS and performed qPCR. Firstly, we confirmed that *PDCD1* expression was dramatically higher in PD-1^high^ ILC2s than PD-1^low^ ILC2s. Next, we assessed the expression of genes related to ILC2 phenotype, function, and immune checkpoint molecules. We found that *CRTH2* and *ST2* expression was more upregulated in PD-1^high^ ILC2s than in PD-1^low^ ILC2s. Since CRTH2 (the receptor for PGD2) and ST2 (the receptor of IL-33) are important functional cytomembrane proteins on ILC2s (40, 41), their upregulation in PD-1^high^ ILC2s implies that PD-1 may enhance the function of ILC2s by boosting the PGD2-CRTH2 and IL-33-ST signaling pathways. We found no difference in the gene expression of the immune checkpoint receptors *CTLA4*, *HAVCR2*, and *LAG3*. Nevertheless, PD-1^high^ ILC2s showed an upregulation of *TIGIT*, which inhibited Th1 cytokine secretion in Treg cells (42) and enhanced IL-4 expression in follicular helper T cells (43), indicating that downregulation of TIGIT might contribute to the upregulation of IL-4 in PD-1^high^ ILC2s. Lastly, we found that IL-4 and IL-13 were highly expressed in PD-1^high^ ILC2s in terms of both mRNA and protein levels, indicating that PD-1^high^ ILC2s may enhance the type 2 immune response by upregulating IL-4 and IL-13 and act as immunosuppressive cells in human NSCLC. In one study, the authors found that PD-1 negatively regulated the function of KLRG1^+^ ILC2s derived from the murine model of worm infection and PBMCs of human HDs *in vitro* (30). However, Batyrova et al. demonstrated that ILC2s from RAG1^-/-^ mice showed stronger capacity of type II cytokine production compared with ILC2s from PD-1^-/-^ xRAG1^-/-^ mice (44). We also found that high expression of PD-1 on ILC2s enhanced IL-4 and IL-13 secretion in NSCLC patients. The reasons for these contradictory findings might be possibly due to: (i) the circumstances surrounding of the immune response to worm infestation and tumor are different; (ii) our data was derived from NSCLC patients and reflected real tumor microenvironment other than those ILC2s induced from HDs *in vitro*. Another study showed that PD-1 inhibited the function of ILC2s and that anti-PD-1 therapy could expand ILC2s and augment anti-tumor immunity (39). However, the present study showed that high expression of PD-1 boosted the function of ILC2, such as enhancing IL4 and IL13 expression, in NSCLC patients. The following are possible reasons for the contradiction: (i) different cancers have distinctive tumor microenvironments; (ii) our study was performed ex vivo which may not reflect the effect of multiple factors *in vivo*, including the ligation of PD-1 and PD-1 ligands and IL-33 activation by ILC2s; and (iii) we speculated that PD-1 upregulation represented activation in the early stage of ILC2s but functioned as an inhibitory factor in later stages, similar to cytotoxic T cells (36). Indeed, a previous study demonstrated that PD-1 expression on ILC2s was upregulated after ILC2 activation by IL-33 in obese mice (45). Thus, in the present study, we found that PD-1 upregulation might be due to ILC2 activation and represented a high-functioning stage in NSCLC ILC2s.

In this study, we found that the phosphorylation levels of AKT and S6 were increased in PD-1^high^ ILC2s, indicating that the PD-1 downstream signaling pathway could be activated in ILC2s obtained from NSCLC patients. However, phosphorylated STAT5, which was reported to be involved in the regulation of cytokine secretion in PD-1 expressing ILC2s in the context of *Nippostrongylus brasiliensis* infection (30), showed no difference in PD-1^high^ ILC2s and PD-1^low^ ILC2s among NSCLC patients. Thus, further studies are needed to elucidate the mechanism by which PD-1 regulates the downstream signaling in ILC2s obtained from NSCLC patients.

TAMs are highly plastic and have functional diversity. M1-like macrophages, which are involved in anti-tumor immunity by secreting pro-inflammatory cytokines, can be polarized by type 1 cytokines such as IFN-γ. Th2-related cytokines (IL-4 and IL-13) regulate M2-like macrophages polarization which play a role in promoting tumor progression by creating an immunosuppressive environment (46). M2-like macrophages can produce cytokines including TGF-β and IL-10 to promote tumor growth (47, 48). In addition, different kind of chemokines from M2-like macrophages including CCL18 and CCL22 mediate immune cells migration to tumor microenvironment (49). In the present study, we found that PD-1^high^ ILC2 culture supernatant enhanced the expression of *MRC1* (encoding CD206, a typical marker of M2 macrophages) and other M2 macrophage related genes (*TGFB1*, *CCL18*, and *ARG1*), and diminished the expression of M1 macrophage related genes (*TNF*, *IL6*, and *CCL5*). Moreover, anti-IL-4 antibody and/or anti-IL-13 antibody could weaken the upregulation of M2 macrophage related genes induced by PD-1^high^ ILC2 culture supernatant. These findings demonstrated that the upregulation of PD-1 on ILC2s could enhance M2-like macrophage polarization from CD14^+^ monocytes by facilitating the production of IL-4 and IL-13. Our findings were in line with some previous reports that IL-4 and IL-13 could mediate M2 macrophage polarization *in vitro* (31, 32).

Dupilumab, a monoclonal antibody against human IL-4 receptor α, inhibits both IL-4 and IL-13 signaling and has been confirmed to be effective in phase 3 clinical trials of asthma and atopic dermatitis (50, 51). IL-4 and IL-13 were upregulated in ILC2s cells, particularly in PD-1^high^ ILC2s in NSCLC patients, and boosted M2-like macrophage polarization, warranting clinical trials of Dupilumab in NSCLC patients in the future.

The present study had some limitations. Firstly, due to the rarity of ILC2s in clinical samples, we could not assess the expression of related proteins using available methods, such as western blot assay. Secondly, we only conducted an exploratory study on PD-1 downstream signaling *via* flow cytometry, and further research is needed to confirm the PD-1 signaling pathway in ILC2s, particularly the molecules involved in the regulation of IL-4 and IL-13 in ILC2s. Thirdly, since there were not enough ILC2s for adaptive transfer assay, we did not ascertain the immunosuppressive function of ILC2s *in vivo*. Thus, well-designed studies are needed to confirm the detailed function of ILC2s in human NSCLC.

## Data Availability Statement

The raw data supporting the conclusions of this article will be made available by the authors, without undue reservation.

## Ethics Statement

The studies involving human participants were reviewed and approved by Ethics Committee of the First Affiliated Hospital of Zhengzhou University. The patients/participants provided their written informed consent to participate in this study.

## Author Contributions

Conception and design: YZ, CS, and CL. Development of methodology: CS, CL, and ZZ. Acquisition of data (acquired and managed patients, provided facilities, etc.): YZ, CS, CL, JS, LW, and YT. Analysis and interpretation of data (e.g. statistical analysis, biostatistics, computational analysis): CS, CL, WY, GQ, YP, and SL. Writing, review, and/or revision of the manuscript: YZ, CS, and CL. Administrative, technical, or material support (i.e. reporting or organizing data, constructing databases): CS, CL, JS, and WY. Study supervision: YZ and ZZ. All authors contributed to the article and approved the submitted version.

## Funding

This work was supported by the National Natural Science Foundation of China (grant no. U1804281 and 81771781), the National Science and Technology Major Project of China (2020ZX09201-009), and the Major public welfare projects in Henan Province (201300310400).

## Conflict of Interest

The authors declare that the research was conducted in the absence of any commercial or financial relationships that could be construed as a potential conflict of interest.

## Supplementary Material

Supplementary Figure 1 Proportions of three subsets in total ILCs from health donors and NSCLC patients. **(A)** The proportions of total ILCs among CD45+ cells from LU-CA (tumor single cell suspension from NSCLC patients, n=5) and LU-N (adjacent tissue cell suspension from NSCLC patients, n=5). **(B)** The distribution of three subsets of ILCs from HD-PB (PBMCs from health donors), LU-PB (PBMCs from NSCLC patients) and LU-CA (tumor single cell suspension from NSCLC patients) via flow cytometry. **(C)** The proportions of ILC2s in LU-CA and LU-N samples (for each group, n=5). **(D)** The proportions of ILC1s in HD-PB, LU-PB and LU-CA samples (for each group, n=20). **(E)** The proportions of ILC3s in HD-PB, LU-PB and LU-CA (for each group, n=20). **(F)** The proportions of different ILC subsets in total ILCs from tumor tissue of NSCLC patients at different stages (for each group, n=20). **(G, H)** The proportions of ILC1s **(G)** and ILC3s **(H)** in total ILCs of LU-CA at different stages (for each group, n=20). NS, not significant, ***P* < 0.01, ****P* < 0.001, *****P* < 0.0001. In **(D, E)**, P values were calculated by one-way ANOVA, post hoc comparisons, Tukey’s test. In **(A, C, G, H)**, P values were calculated by non-paired two-tailed Student t-test.

Supplementary Figure 2 Gene expression of surface markers and functional molecules of ILC2s from HDs and NSCLC patients. The mRNA expression of surface marker genes **(A)** and functional molecules genes **(B)** of ILC2s sorted from HD-PB, LU-PB and LU-CA samples was tested by qPCR (for each group, n=18). ILC2s were marked as CD45+Lin-CD127+CRTH2+. HD-PB: PBMCs from health donors, LU-PB: PBMCs from NSCLC patients, and LU-CA: tumor single cell suspension from NSCLC patients. NS, not significant, **P* < 0.05, ***P* < 0.01, ****P* < 0.001, *****P* < 0.0001. P values were calculated by one-way ANOVA, post hoc comparisons, Tukey’s test.

Supplementary Figure 3 The expression of immune checkpoint molecules in ILC2s. **(A)** Gene expression of immune checkpoint molecules of ILC2s from health donors and NSCLC patients. ILC2s were sorted by FACS. The mRNA expression of other immune checkpoint genes in ILC2s from HD-PB, LU-PB and LU-CA samples was tested by qPCR (for each group, n=18). **(B)** The proportions of PD-1 expression in ILC2s from LU-CA and LU-N samples (for each group, n=5). NS, not significant, **P* < 0.05. In **(A)**, *P* values were calculated by one-way ANOVA, post hoc comparisons, Tukey’s test. In **(B)**, *P* values were calculated by non-paired two-tailed Student t-test.

Supplementary Figure 4 Gene expression of surface markers, immune checkpoint molecules and functional molecules of PD-1high and PD-1low ILC2s from tumor tissue of NSCLC patients. PD-1high ILC2s and PD-1low ILC2s were marked and sorted as described in **Figure 4**. **(A)** The mRNA expression of surface marker genes of ILC2s sorted from HD-PB, LU-PB and LU-CA samples (n=26). **(B)** The mRNA expression of other immune checkpoint genes in ILC2s from HD-PB, LU-PB and LU-CA samples. **(C)** The mRNA expression of other functional genes in ILC2s from HD-PB, LU-PB and LU-CA samples. NS, not significant, **P* < 0.05. P values were calculated by paired two-tailed Student t-test.

Supplementary Figure 5 PD-1high ILC2s boosted M2 related genes expression and downregulated M1 related genes through secreting IL-4 and IL-13. **(A, B)** PD-1high ILC2s culture supernatant downregulated M1 related genes (TNF, IL6 and CCL5) expression **(A)** and upregulated M2 related genes (TGFB1, CCL18 and ARG1) expressions **(B)**. **(C)** Anti-IL-4 antibody and/or anti-IL-13 antibody weakened the upregulation of M2 related genes expression in CD14+ cells treated by PD-1high ILC2s culture supernatant. NS, not significant, **P* < 0.05, ***P* < 0.01, ****P* < 0.001, *****P* < 0.0001. P values were calculated by one-way ANOVA, post hoc comparisons, Tukey’s test.
