# Supplementary material for: PD-1 Affects the Immunosuppressive Function of Group 2 Innate Lymphoid Cells in Human Non-Small Cell Lung Cancer
Source: Front Immunol. 2021 Jun 14;12:680055. doi: 10.3389/fimmu.2021.680055 (PMC8237944; doi:10.3389/fimmu.2021.680055)
Supplement: Supplementary file 6 [file DataSheet_1.docx]

**Supplementary Figure Legends**

**Supplementary Figure 1**. Proportions of three subsets in total ILCs from health donors and NSCLC patients. (**A**) The proportions of total ILCs among CD45+ cells from LU-CA (tumor single cell suspension from NSCLC patients, n=5) and LU-N (adjacent tissue cell suspension from NSCLC patients, n=5). (**B**) The distribution of three subsets of ILCs from HD-PB (PBMCs from health donors), LU-PB (PBMCs from NSCLC patients) and LU-CA (tumor single cell suspension from NSCLC patients) via flow cytometry. (**C**) The proportions of ILC2s in LU-CA and LU-N samples (for each group, n=5). (**D**) The proportions of ILC1s in HD-PB, LU-PB and LU-CA samples (for each group, n=20). (**E**) The proportions of ILC3s in HD-PB, LU-PB and LU-CA (for each group, n=20). (**F**) The proportions of different ILC subsets in total ILCs from tumor tissue of NSCLC patients at different stages (for each group, n=20). (**G, H**) The proportions of ILC1s (**G**) and ILC3s (**H**) in total ILCs of LU-CA at different stages (for each group, n=20). NS, not significant, **, *P*<0.01, ***, *P*<0.001, ****, *P*<0.0001. In Figure **D** and **E**，*P* values were calculated by one-way ANOVA, post hoc comparisons, Tukey’s test. In Figure **A**, **C**, **G,** and **H**，*P* values were calculated by non-paired two-tailed Student t-test.

**Supplementary Figure 2**. Gene expression of surface markers and functional molecules of ILC2s from HDs and NSCLC patients. The mRNA expression of surface marker genes (**A**) and functional molecules genes (**B**) of ILC2s sorted from HD-PB, LU-PB and LU-CA samples was tested by qPCR (for each group, n=18). ILC2s were marked as CD45^+^Lin^-^CD127^+^CRTH2^+^. HD-PB: PBMCs from health donors, LU-PB: PBMCs from NSCLC patients, and LU-CA: tumor single cell suspension from NSCLC patients. NS, not significant, *, *P*<0.05, **, *P*<0.01, ***, *P*<0.001, ****, *P*<0.0001. *P* values were calculated by one-way ANOVA, post hoc comparisons, Tukey’s test.

**Supplementary Figure 3**. The expression of immune checkpoint molecules in ILC2s. (**A**) Gene expression of immune checkpoint molecules of ILC2s from health donors and NSCLC patients. ILC2s were sorted by FACS. The mRNA expression of other immune checkpoint genes in ILC2s from HD-PB, LU-PB and LU-CA samples was tested by qPCR (for each group, n=18). (**B**) The proportions of PD-1 expression in ILC2s from LU-CA and LU-N samples (for each group, n=5). NS, not significant, *, *P*<0.05. In Figure **A**, *P* values were calculated by one-way ANOVA, post hoc comparisons, Tukey’s test. In Figure **B**, *P* values were calculated by non-paired two-tailed Student t-test.

**Supplementary Figure 4**. Gene expression of surface markers, immune checkpoint molecules and functional molecules of PD-1^high^ and PD-1^low^ ILC2s from tumor tissue of NSCLC patients. PD-1^high^ ILC2s and PD-1^low^ ILC2s were marked and sorted as described in Figure 4. (**A**) The mRNA expression of surface marker genes of ILC2s sorted from HD-PB, LU-PB and LU-CA samples (n=26). (**B**) The mRNA expression of other immune checkpoint genes in ILC2s from HD-PB, LU-PB and LU-CA samples. (**C**) The mRNA expression of other functional genes in ILC2s from HD-PB, LU-PB and LU-CA samples. NS, not significant, *, *P*<0.05. *P* values were calculated by paired two-tailed Student t-test.

**Supplementary Figure 5**. PD-1^high^ ILC2s boosted M2 related genes expression and downregulated M1 related genes through secreting IL-4 and IL-13. (**A-B**) PD-1^high^ ILC2s culture supernatant downregulated M1 related genes (*TNF*, *IL6* and *CCL5*) expression (**A**) and upregulated M2 related genes (*TGFB1*, *CCL18* and *ARG1*) expressions (**B**). (**C**) Anti-IL-4 antibody and/or anti-IL-13 antibody weakened the upregulation of M2 related genes expression in CD14^+^ cells treated by PD-1^high^ ILC2s culture supernatant. NS, not significant, *, *P*<0.05, **, *P*<0.01, ***, *P*<0.001, ****, *P*<0.0001. *P* values were calculated by one-way ANOVA, post hoc comparisons, Tukey’s test.
