# Supplementary material for: PD-1 Affects the Immunosuppressive Function of Group 2 Innate Lymphoid Cells in Human Non-Small Cell Lung Cancer
Source: Front Immunol. 2021 Jun 14;12:680055. doi: 10.3389/fimmu.2021.680055 (PMC8237944; doi:10.3389/fimmu.2021.680055)
Supplement: Supplementary file 7 [file Table_1.docx]

**Supplementary Table S1.** Clinical pathological characteristics of patients with NSCLC

| **Patient characteristics** | | **NSCLC patients**  **(n=70)**  **n (%)** |
| --- | --- | --- |
| Gender | Male | 38 (54.3 %) |
|  | Female | 32 (45.7 %) |
| Age (years) | < 60 | 31 (44.3 %) |
|  | ≥ 60 | 39 (55.7 %) |
| Smoking history | Yes | 33 (47.1 %) |
|  | No | 37 (52.9 %) |
| Histological type | Squamous carcinoma | 33 (47.1 %) |
|  | Adenocarcinoma | 37 (52.9 %) |
| Histological grade | I | 11 (15.7 %) |
|  | II | 23 (32.9 %) |
|  | III | 36 (51.4 %) |
| TNM stage | I-II | 30 (42.9 %) |
|  | III-IV | 40 (57.1 %) |
| Lymph node metastasis | Yes | 38 (54.3 %) |
|  | No | 32 (45.7 %) |
