# Supplementary material for: PD-1 Affects the Immunosuppressive Function of Group 2 Innate Lymphoid Cells in Human Non-Small Cell Lung Cancer
Source: Front Immunol. 2021 Jun 14;12:680055. doi: 10.3389/fimmu.2021.680055 (PMC8237944; doi:10.3389/fimmu.2021.680055)
Supplement: Supplementary file 8 [file Table_2.docx]

**Supplementary Table S2.** Primers for quantitative real-time PCR

| Gene Name | Primer Forward | Primer Reverse | PCR product |
| --- | --- | --- | --- |
| *AREG* | CCAAAACAAGACGGAAAGTGA | TGTTACTGCTTCCAGGTGCTC | 175bp |
| *ARG1* | TGGACAGACTAGGAATTGGCA | CCAGTCCGTCAACATCAAAACT | 102bp |
| *CCL5* | CTCCCCATATTCCTCGGACA | GTTGATGTACTCCCGAACCC | 190bp |
| *CCL18* | TACCTCCTGGCAGATTCCAC | CCCACTTCTTATTGGGGTCA | 131bp |
| *CRTH2* | CTGGGCAAGTGCCTCCTAAG | CAACGAGTTGTCCAATGCGG | 93bp |
| *CTLA4* | CATGATGGGGAATGAGTTGACC | TCAGTCCTTGGATAGTGAGGTTC | 92bp |
| *GADPH* | GCACCGTCAAGGCTGAGAAC | TGGTGAAGACGCCAGTGGA | 138bp |
| *HAVCR2* | CTGCTGCTACTACTTACAAGGTC | GCAGGGCAGATAGGCATTCT | 75bp |
| *IL13* | GGTCAACATCACCCAGAACC | GATTCCAGGGCTGCACAGTA | 99bp |
| *IL4* | TCCGATTCCTGAAACGGCTC | TGGTTGGCTTCCTTCACAGG | 79bp |
| *IL5* | CGAACTCTGCTGATAGCCAA | CAGTACCCCCTTGCACAGTT | 130bp |
| *IL6* | GAACCTTCCAAAGATGGC | CAAATCTGTTCTGGAGGT | 131bp |
| *IL7R* | CTCCAACCGGCAGCAATGTAT | AGATGACCAACAGAGCGACAG | 193bp |
| *IL9* | CTTCTGGCCATGGTCCTTAC | CATGGTCTGGTGCAGTTGTC | 200bp |
| *KIT* | ACTTGAGGTTTATTCCTGACCCC | GCAGACAGAGCCGATGGTAG | 78bp |
| *LAG3* | GCCTCCGACTGGGTCATTTT | CTTTCCGCTAAGTGGTGATGG | 131bp |
| *MRC1* | GGGTTGCTATCACTCTCTATGC | TTTCTTGTCTGTTGCCGTAGTT | 126bp |
| *PDCD1* | CCAAGGCGCAGATCAAAGAGA | AGGACCCAGACTAGCAGCA | 175bp |
| *ST2* | ATGGGGTTTTGGATCTTAGCAAT | CACGGTGTAACTAGGTTTTCCTT | 138bp |
| *TGFB1* | GGCCAGATCCTGTCCAAGC | GTGGGTTTCCACCATTAGCAC | 201bp |
| *TIGIT* | TCTGCATCTATCACACCTACCC | CCACCACGATGACTGCTGT | 162bp |
| *TNF* | GAGGCCAAGCCCTGGTATG | CGGGCCGATTGATCTCAGC | 91bp |
